# Cytogenetic analysis in the *incertae sedis* species *Astyanax altiparanae* Garutti and Britzki, 2000 and *Hyphessobrycon eques* Steindachner, 1882 (Characiformes, Characidae) from the upper Paraná river basin

**DOI:** 10.3897/CompCytogen.v6i1.1873

**Published:** 2012-02-14

**Authors:** Emanuel R. M. Martinez, Anderson L. Alves, Sara M. Silveira, Fausto Foresti, Claudio Oliveira

**Affiliations:** 1 Departamento de Morfologia, Laboratório de Biologia e Genética de Peixes, Universidade Estadual Paulista, Instituto de Biociências, Bairro Rubião Jr., s/n, 18618-000, Botucatu, SP, Brazil; 2 Departamento de Biologia, Universidade Estadual Paulista, Av. 24A, 1515, Bairro Bela Vista, 13506-900, Rio Claro, SP, Brazil

**Keywords:** Ag-NOR, Chromomycin A_3_, chromosomes, evolution, Neotropical fish

## Abstract

Cytogenetic analyses were accomplished in two populations of *Astyanax altiparanae* Garutti & Britzki, 2000 and one population of *Hyphessobrycon eques* Steindachner, 1882, considered *incertae sedis* in Characidae family. Two populations of *Astyanax altiparanae* (Mogi-Guaçu and Tietê rivers) presented 2n=50, with the same karyotype formula: 6M+12SM+20ST+12A (FN=88). *Hyphessobrycon eques* from Capivara river presented 2n=52 and karyotype formula 14M+16SM+4ST+18A (FN=86). In each karyotype, the nucleolus organizer regions were detected at the end of the short arm of a single medium-sized subtelocentric chromosome. The Chromomycin A_3 _(CMA_3_) marking is coincident for the NORs in chromosomes of the two species and present additionally in two different chromosomes of *Astyanax altiparanae* thus showinginterpopulation differences in this species. In *Hyphessobrycon eques*, weak heterochromatic blocks in the position of centromeres and telomeres of most chromosomes and negative C-banding for the NOR bearing chromosome were visualized. The obtained results contribute both to the understanding of karyotype evolution of these species and to the clarifying their phylogenetic relationships.

## Introduction

The neotropical freshwater ichthyofauna is quite rich, including 71 families and more than 4,500 species known to be valid, according to the latest surveys (Reis et al. 2003, Nelson 2006, Buckup et al. 2007). The Characiformes are exclusively freshwater fish, distributed in America and Africa with highest diversity in the major Neotropical basins (Buckup 1998). Currently, this order comprises 1,674 valid species in 270 genera (Nelson 2006), a number probably underestimated (Vari 1998).

*Astyanax* Baird and Girard, 1854 and *Hyphessobrycon* Eigenmann, 1908 are genera from family Characidae with wide distribution throughout Central and South America, and previously placed in subfamily Tetragonopterinae (Géry 1977). Recently these groups were considered as *incertae sedis* in family Characidae together with about 100 genera (Lima et al.2003, Nelson 2006). Regarding the phylogenetic relationship between the genera considered *incertae sedis*, Mirande (2009) proposed a hypothesis that suggests a close relationship between *Astyanax* and *Hyphessobrycon*, forming, along with other genera, the clade *Astyanax*.

*Astyanax* includes about 100 species, commonly known as lambari and piaba (Lima et al. 2003, Nelson 2006). Reviewing some species of *Astyanax*, Garutti and Britzki (2000) described *Astyanax altiparanae*, a new species formally presented as *Astyanax bimaculatus* Linnaeus, 1758, for the upper Paraná river basin. *Astyanax altiparanae* presents a black humeral spot horizontally oval, two brown vertical bars located in the humeral region, and a black diamond spot at caudal peduncle that extends to the tip of median caudal rays (Lima et al. 2003).

The genus *Hyphessobrycon* with approximately 90 species is characterized by an interrupted lateral line, reaching up to 60 mm in total length, and some species present a remarkable color that may interest the aquarists (Lima et al. 2003, Nelson 2006). *Hyphessobrycon eques* are commonly known as matogrossinhos, with distribution in the South American river basins, also in *La Plata* basin (Paraná-Paraguai-Uruguai-Prata) and in rivers of Amazonas basin (Lima et al. 2003, Nelson 2006).

Cytogenetic analysis of representatives of the genus *Astyanax* reveals that diploid numbers range from 2n=36 in *Astyanax schubarti* Britski, 1964 (Morelli et al. 1983) to 2n=50 chromosomes in *Astyanax paranae* Eigenmann, 1914 (Vicari et al. 2008). According to Galetti et al. (1994) the karyotype data from the genus *Astyanax* present a chromosomal variability between different species and characterize a karyotypic heterogeneity in evolution of this group due to structural chromosomal rearrangements, mainly of Robertsonian type. Additional levels of chromosomal evolution may be uncovered in intraspecific studies and with the use of various chromosome techniques. About twenty populations of *Astyanax altiparanae* studied to date (Table 1) present 2n=50 chromosomes with differences in their karyotypic formula and in the number and position of nucleolar organizer regions (NOR) in the chromosomes (Fernandes and Martins-Santos 2004, Neto et al. 2009) (Table 1).

**Table 1.** Summary of cytogenetic data from Brazilian populations of *Astyanax altiparanae* and *Hyphessobrycon* spp. 2n - diploid number; M - metacentric; SM - submetacentric; ST - subtelocentric; A - acrocentric; FN = fundamental number; n-NORs - number of chromosomes with silver stained nucleolar organizer regions. * - cited as *Astyanax bimaculatus* Linnaeus, 1758. Location’s list follows a geographical order.

**Species**	**Location**	**2n**	**Karyotype**	**FN**	n**-NORs**	**Reference**
*Astyanax altiparanae* Garutti & Britzki, 2000	Iguaçu river, Curitiba, PR (Iguaçu river basin)	50	6M+30SM+8ST+6A	94	2	Domingues et al. (2007)
	Jordão river, Manguerinha, PR (Iguaçu river basin)	50	6M+28SM+8ST+8A	92	2-4	Neto et al. (2009)
	Índios river, Cianorte, PR (Ivaí river basin)	50	6M+30SM+4ST+10A	90	10	Fernandes and Martins-Santos (2004)
	Tatupeba river, Maringá, PR (Ivaí river basin)	50	6M+26SM+6ST+12A	88	3	Fernandes and Martins-Santos (2006)
	*Meia Ponte river, Goiânia, GO (Meia Ponte river basin)	50	26M+24A	76	-	Fernandes and Martins-Santos (2006)
	Feijão stream, São Carlos, SP (Mogi-Guaçu river basin)	50	6M+30SM+8ST+6A	94	1-3	Neto et al. (2009)
	*Mogi-Guaçu river, Pirassununga, SP (Mogi-Guaçu river basin)	50	10M+24SM+4ST+12A	88	-	Morelli et al. (1983)
	Mogi-Guaçu river, Pirassununga, SP (Mogi-Guaçu river basin)	50	6M+12SM+20ST+12A	88	2	Present study
	Paraná river, Porto Rico, PR (Paraná river basin)	50	6M+26SM+6ST+12A	88	2	Fernandes and Martins-Santos (2006)
	Claro river, Tamarana, PR (Paranapanema river basin)	50	10M+26SM+4ST+10A	90	1-4	Pacheco et al. (2001)
	Claro river, Tamarana, PR (Paranapanema river basin)	50	10M+24SM+4ST+12A	88	1-4	Pacheco et al. (2001)
	Claro river, Tamarana, PR (Paranapanema river basin)	50	10M+22SM+4ST+14A	86	1-4	Pacheco et al. (2001)
	Paranapanema river, Salto Grande, SP (Paranapanema river basin)	50	10M+22SM+6ST+12A	88	-	Fernandes and Martins-Santos (2006)
	Keçaba river, Maringá, PR (Pirapó river basin)	50	6M+26SM+6ST+12A	88	1	Fernandes and Martins-Santos (2006)
*Astyanax altiparanae* Garutti & Britzki, 2000	Maringá river, Maringá, PR (Pirapó river basin)	50	6M+26SM+6ST+12A	88	3	Fernandes and Martins-Santos (2006)
	*São Francisco river, MG (São Francisco river basin)	50	-	-	-	Carvalho et al. (2002b)
	Tibagi river, Ponta Grossa, PR (Tibagi river basin)	50	6M+28SM+8ST+8A	92	2-3	Domingues et al. (2007)
	*Jurumirim river, SP (Tietê river basin)	50	-	-	-	Carvalho et al. (1998)
	Pântano stream, São Carlos, SP (Tietê river basin)	50	6M+28SM+4ST+12A	88	1-2	Neto et al. (2009)
	Tietê river, Penápolis, SP (Tietê river basin)	50	6M+12SM+20ST+12A	88	2	Present study
*Hyphessobrycon anisitsi* Eigenmann, 1907	Piracuama river (Paraíba do Sul river basin)	50	6M+16SM+12ST+16A	84	4	Centofante et al. (2003)
*Hyphessobrycon anisitsi* Eigenmann, 1907	Perdizes stream (Paraná river basin)	50	6M+16SM+12ST+16A	84	3	Centofante et al. (2003)
*Hyphessobrycon flammeus* Myers, 1924	Paraná river (Paraná river basin)	52	18M,SM+32ST+2A	102	-	Fernandes and Martins-Santos (2006)
*Hyphessobrycon reticulatus* Ellis, 1911	Juquiá river, São Lourenço da Serra, SP (Paraná river basin)	50	14M+20SM+16ST	100	2	Carvalho et al. (2002a)
*Hyphessobrycon scholzei* Ahl, 1937	Perdizes stream (Paraná river basin)	50	8M+20SM+8ST+14A	86	-	Fernandes and Martins-Santos (2006)
*Hyphessobrycon griemi* Hoedeman, 1957	Itimirim river, Iguape, SP, Iguape river basin (Ribeira river basin)	48	-	-	-	Carvalho et al. (2002b)
*Hyphessobrycon herbertaxelrodi* Géry, 1961	Itimirim river, Iguape, SP, Iguape river basin (Ribeira river basin)	52	10M,SM+42ST,A	-	-	Fernandes and Martins-Santos (2006)
*Hyphessobrycon reticulatus* Ellis, 1911	Itimirim river, Iguape, SP, Iguape river basin (Ribeira river basin)	50	-	-	-	Carvalho et al. (2002b)
*Hyphessobrycon eques* Steindchner, 1882	Capivara river, Botucatu, SP (Tietê river basin)	52	14M+16SM+4ST+18A	86	2	Present study

Little is known about the cytogenetic patterns for *Hyphessobrycon*, where only a few species have been karyotyped (Table 1), and for many of them, only the haploid number is known (Sheel 1973). Nevertheless, the chromosome number is variable among the species, between 2n=48 for *Hyphessobrycon griemi* Hoedeman, 1957 (Carvalho et al. 2002a) and 2n=52 for *Hyphessobrycon herbertaxelrodi* Géry, 1961 (Arefjev 1990) (Table 1). Karyotypic data for *Hyphessobrycon eques* are not available in literature.

In the present study, we compare the karyotypes of two populations of *Astyanax altiparanae* and one of *Hyphessobrycon eques* aiming to contribute to the increase of knowledge about the patterns of diversity and evolution of karyotype in this *incertae sedis* group of Characidae.

## Materials and methods

Specimens from two populations of *Astyanax altiparanae* and one of *Hyphessobrycon eques* were collected in streams from the upper Paraná river basin (Fig. 1). The individuals were anesthetized with benzocaine (5%) and then sacrificed for subsequent cytogenetic analysis. The processed specimens were fixed in 10% formalin and stored in 70% alcohol for further taxonomic studies. The preserved specimens were placed in the collection of fish from Laboratório de Biologia e Genética de Peixes (LBP), Departamento de Morfologia do Instituto de Biociências da UNESP, campus de Botucatu. Their deposit numbers are indicated below.

**Figure l.**
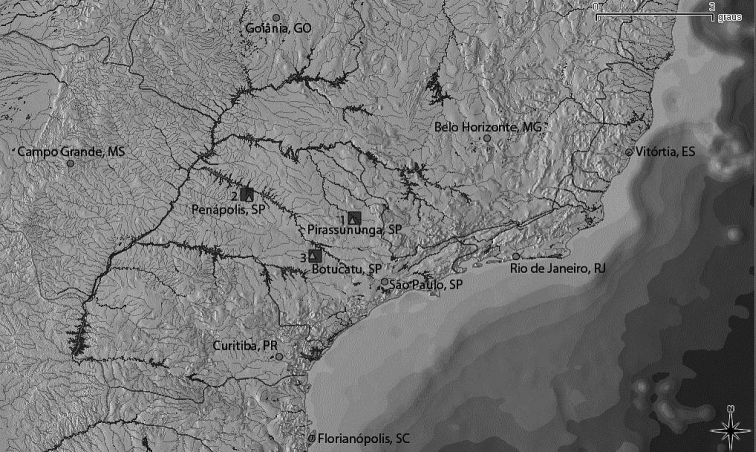
Map of the collection sites (squares) for the *Astyanax altiparanae* (**1, 2**) and *Hyphessobrycon eques* (**3**) in three rivers of the upper Paraná basin, São Paulo State (SP). Triangles refer to the neighboring cities and circles to the capitals of the states.

The following specimens were karyotyped: six males and four females of *Astyanax altiparanae* from the Mogi-Guaçu river, Pirassununga, SP., Brazil (Mogi-Guaçu river basin, site 1 in the map, GPS: 21°55'37.6"S, 47°22'04.4"W) with number 1142 (LBP); four males and two females of *Astyanax altiparanae* from the Tietê river, Penápolis, SP., Brazil (Tietê river basin, site 2, GPS: 21°18'46.1"S, 50°08'26.4"W) with number 2690 (LBP); and three males and two females of *Hyphessobrycon eques* from the Capivara river, Botucatu, SP., Brazil (Tietê river basin, site 3, GPS: 22°53'57.6"S, 48°23'11.4"W) with number 2337 (LBP) (Fig. 1).

**Figure 2.**
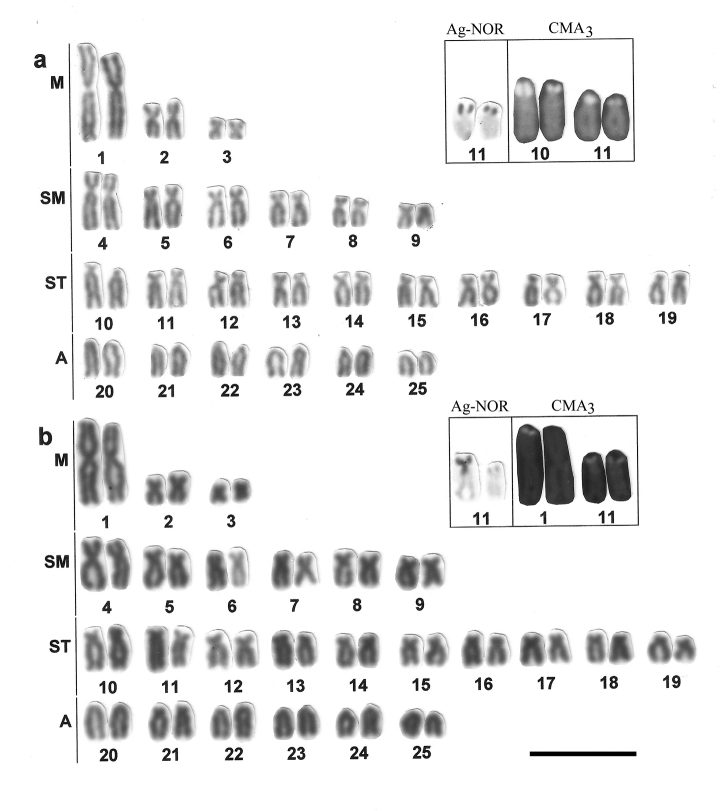
Karyograms showing chromosome morphology with the results of NOR-silver staining and Chromomycin A_3 _(CMA_3_) treatment (in a frame) on chromosomes of *Astyanax altiparanae* from Mogi-Guaçu river (**a**) and Tietê river (**b**). Bar = 5µm.

**Figure 3.**
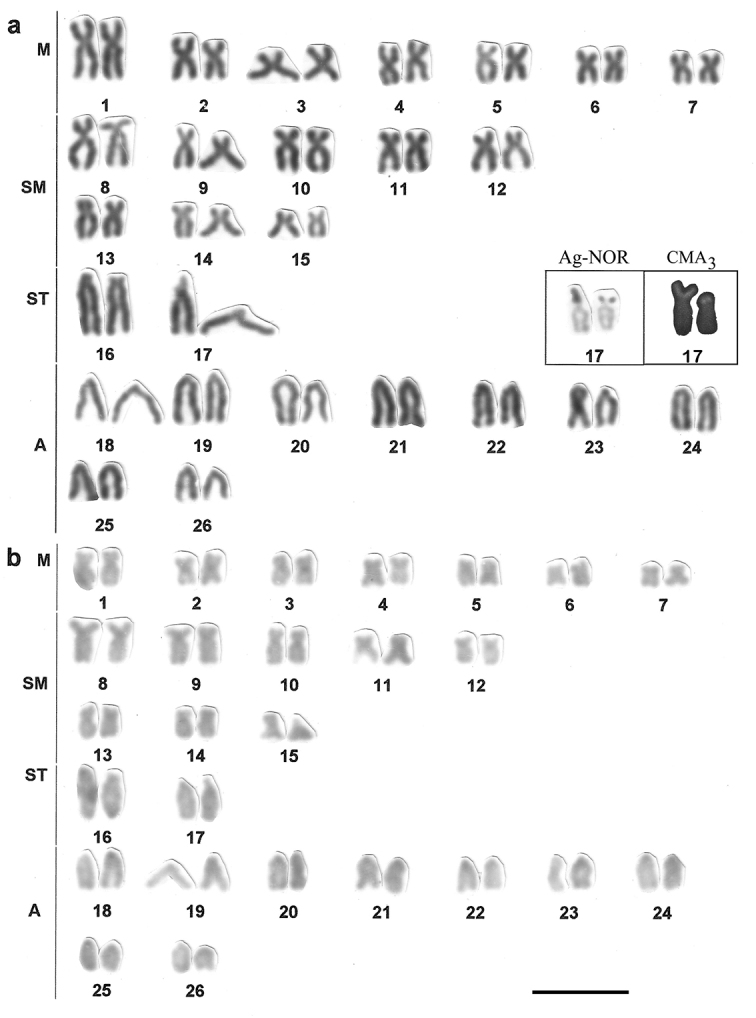
Karyotype of *Hyphessobrycon eques* showing (**a**) chromosome morphology with the results of NOR-silver and Chromomycin A_3 _(CMA_3_) staining in a frame, and (**b**) C-banding in fish individuals collected in the Capivara river. Bar = 5µm.

Metaphase chromosomes were studied on slide preparations made from kidney through the common air dryingtechnique (Foresti et al. 1981), with the followed detection of the nucleolus organizer regions by the silver impregnation technique (Ag-NOR) from Howell and Black (1980), C-banding by the method of Sumner (1972), and flouorescent chromosome staining with Chromomycin A_3_ (CMA_3_) according to Schweiser (1976). The chromosome morphology was established based on the arm proportions about the centromere, as proposed by Levan et al. (1964), and the chromosome nomenclature commonly attributed to fish as metacentric (M), submetacentric (SM), subtelocentric (ST) and acrocentric (A) was used. Grouped correspondingly, the chromosomes were arranged in the hand constructed photo-karyograms of 3 fish populations studied (Figs 2, 3).

## Results and discussion

Among the populations of *Astyanax altiparanae* studied to date, all presented a diploid number of 50 chromosomes, and that is true for the two populations examined in the present study (Table 1). The karyogram of the species contains one big and two small metacentric pairs, a large group of submeta-subtelocentrics and not less than 6 acrocentric pairs (Figs 2a, b). The chromosome morphology did not show populational variations in samples from Mogi-Guaçu river and Tietê river in our data, which share the karyotype formula 6M+12SM+20ST+12A, and fundamental number is accordingly 88.

This chromosomal uniformity is, however, not common for populations from distinct hydrographic basins and even within the same basin, as other populations of the rivers Tiete and Mogi-Guacu basins show considerable variation (Morelli et al. 19983, Neor et al. 2009) (see Table 1).

The intraspecific variety of chromosome formulae in this case is due to the variable content of each morphological group, from M to A, which interpretation may be difficult, however, without chromosome specific markers, still poorly available in ordinary fish cytogenetics. It should be stressed for this species that the presence of the big metacentric (pair M1) seems to be not only the common karyotype feature of species specific significance for *Astyanax altiparanae*,but also a provisional phylogenetic marker. Taking into account the possibility of appearance of such a large bi-armed chromosome through Robertsonian fusion, it might focus to the karyotype relation between taxa under this study differing in 2n. Arm chromosome variation within and between the karyotypes might be caused by intrachromosomal changes such as pericentric inversion, centromeric shift, heterochromatin or NOR position.

The chromosome number is variable among the *Hyphessobrycon* species, ranging from 2n=52 chromosomes in *Hyphessobrycon herbertaxelrodi* (Arefjev 1990) to 2n=48 in *Hyphessobrycon griemi* (Carvalho et al. 2002b). Cytogenetic study of *Hyphessobrycon eques* reveals a diploid number of 52 chromosomes and a karyotype formula with 14M+16SM+4ST+18A and FN=86. There are 9 acrocentric and 2 subtelocentric pairs in the karyogram and a large group of medium-sized submeta-metacentrics (Fig. 3). This is the first karyotype presentation of the species (Table 1).

Impregnation by silver nitrate reveals a single NOR location in a subtelocentric chromosome for populations of *Astyanax altiparanae* and for the species *Hyphessobrycon eques* (Figs 2, 3, Table 1). In *Astyanax altiparanae*, the NOR marks are presented on a short arm of the subtelocentric pair 11 (Figs 2a, b, Table 1). In *Hyphessobrycon eques*, the similar NOR bearing subtelocentric corresponds to the chromosome 17 in the species karyogram (Fig. 3).

The treatment with fluorochrome Chromomycin A_3_ (CMA_3_) was used to evidence NOR as regions associated with GC-rich DNA (Schmid and Guttenbach 1989). The Chromomycin A_3_ treatment of chromosome preparations of *Astyanax altiparanae* from Mogi-Guaçu river revealed marks in two chromosome pairs, one coincident with the NOR location in the pair 11, and the other on a short arm of the morphologically similar subtelocentrics of the pair 10 (Fig. 2a). However, in specimens from the Tietê river population, besides the coincident marking with the NOR bearing pair 11, a mark on one of the homologs of the largest pair (M1), was also detected (Fig. 2b).

NOR pattern variation has been reported for this species, from single to multiple NORs, which may characterize as intra- as inter-population variation in NOR location and chromosome morphology as well (Pacheco et al. 2001, Domingues et al. 2007, Neto et al. 2009). In our materials, the Chromomycin A_3 _treatment data may suggest on possibility of the activity of extra number NORs above the single NOR pattern coincident with sylver staining in the upper Paraná populations studied.

In *Hyphessobrycon* species, too, there is a great variation of NORs appearing as single sites (Carvalho et al. 2002a) or multiple marked sites on chromosomes of the species (Centofante et al. 2003), that strengths the hypothesis of intensive chromosomal rearrangements in the group. C-banding identified in the genus, namely in *Hyphessobrycon reticulatus* Ellis, 1911 (Carvalho et al. 2002a) and *Hyphessobrycon anisitsi* Eigenmann, 1907 (Centofante et al. 2003), appeared as small pericentromeric blocks in all chromosomes of the karyotype. In *Hyphessobrycon anisitsi*, however, some chromosomes presented also terminal heterochromatic blocks, that was considered as indication on interpopulation differentiation. In view of uncertain chromosome identification, these data remain a preliminary information for further analyses only.

According to Mirande (2009), the *Astyanax* clade includes (along with all included species of *Astyanax*) *Markiana* Eigenmann, 1907, *Psellogrammus* Eigenmann, 1908, probably *Ctenobrycon* Eigenmann, 1908 and some *Bryconamericus* Eigenmann, 1907 and *Hyphessobrycon* taxa, suggesting that this highly diverse genus could be diagnosed as monophyletic with relatively few changes in its composition. Nevertheless the observed karyotype variations and poor supporting chromosome details, we could suggest rather close phylogenetic interrelation from the comparison of karyotypes of the two genera, *Astyanax* and *Hyphessobrycon*. It follows from an assumption of a Robertsonian change between the generic karyotypes viewed through changes of their morphology and 2n at maximal generic levels (50, 52) and proposedly common cytogenetic tools for multiple chromosomal differentiation of populations and species (NOR and C-banding patterns) though parallelisms cannot be excluded.
